# Manchester Royal Infirmary

**Published:** 1924-10

**Authors:** Herbert J. Dafforne

**Affiliations:** Secretary. Ancoats Hospital, Manchester


### Manchester Royal Infirmary.

Sir,—On page 288 of this month's Hospital and Health Review, under the heading " Manchester Victoria University," you state that the Manchester Royal Infirmary has 1,663 beds. This is, of course, an error and should be 663 beds.

Herbert J. Dafforne,

Ancoats Hospital, Manchester. Secretary.

